# Novel evolutionary dynamics of small populations in breast cancer adjuvant and neoadjuvant therapy

**DOI:** 10.1038/s41523-021-00230-y

**Published:** 2021-03-11

**Authors:** Yael Artzy-Randrup, Tamir Epstein, Joel S. Brown, Ricardo L. B. Costa, Brian J. Czerniecki, Robert A. Gatenby

**Affiliations:** 1 Department of Theoretical and Computational Ecology, IBED, University of Amsterdam, Amsterdam, The Netherlands; 2 Institute of Advanced Study, University of Amsterdam, Amsterdam, The Netherlands; 3 Integrated Mathematical Oncology Department, H. Lee Moffitt Cancer Center and Research Institute, Tampa, FL USA; 4 Cancer Biology and Evolution Program, H. Lee Moffitt Cancer Center and Research Institute, Tampa, FL USA; 5 Breast Oncology Department, H. Lee Moffitt Cancer Center and Research Institute, Tampa, FL USA; 6 Diagnostic Imaging Department, H. Lee Moffitt Cancer Center and Research Institute, Tampa, FL USA

**Keywords:** Cancer models, Breast cancer, Cancer therapeutic resistance

## Abstract

Disseminated cancer cells (DCCs) are detected in the circulation and bone marrow of up to 40% of breast cancer (BC) patients with clinically localized disease. The formation of metastases is governed by eco-evolutionary interactions of DCCs with the tissue during the transition from microscopic populations to macroscopic disease. Here, we view BC adjuvant and neoadjuvant treatments in the context of small population extinction dynamics observed in the Anthropocene era. Specifically, the unique eco-evolutionary dynamics of small asexually reproducing cancer populations render them highly vulnerable to: (1) environmental and demographic fluctuations, (2) Allee effects, (3) genetic drift and (4) population fragmentation. Furthermore, these typically interact, producing self-reinforcing, destructive dynamics—termed the Extinction Vortex—eradicating the population even when none of the perturbations is individually capable of causing extinction. We propose that developing BC adjuvant and neoadjuvant protocols may exploit these dynamics to prevent recovery and proliferation of small cancer populations during and after treatment—termed “Eco-evolutionary rescue” in natural extinctions. We hypothesize more strategic application of currently available agents based on the extinction vulnerabilities of small populations could improve clinical outcomes.

## Introduction

Adjuvant or neoadjuvant therapy is commonly used to reduce the risk of subsequent metastases in “high risk”^1^ primary breast cancers (BC) when no metastatic sites are clinically evident. The specific treatment is largely determined by the clinical subtype defined by the expression of hormone and HER2 receptors. In general, clinical benefits are significant but not universal. For example, six 4-week cycles of cyclophosphamide, methotrexate, prednisone, and fluorouracil increased 3-year survival from 69 to 84% in a group of over 500 women following resection of high risk, node-negative primary BC^2^.

Similarly, in HER2-positive BC, current multi-agent chemotherapy, and targeted therapies, when applied in the adjuvant or neoadjuvant setting virtually eliminate the development of metastases in small, node-negative primary cancers^3^. In more advanced stage disease, adjuvant and neoadjuvant, treatments only moderately reduce the probability of subsequent metastases.

These are tantalizing data that show eradication of small BC populations is possible, yet neoadjuvant/adjuvant therapy often fails suggesting alternative strategies for application of current treatment agents may improve outcomes. We propose that adjuvant and neoadjuvant treatments to prevent metastases can be viewed as an “Anthropogenic extinction”^4,5^. Treatments fail to cause extinction in a significant fraction of patients because some members of the populations survive (“evolutionary rescue”^6^) and then proliferate to produce fatal metastatic disease. Furthermore, particularly in BC, some surviving cells may remain quiescent suggesting a steady state in which births equal deaths. However, with time these may be subject to perturbations owing to aging, injury of the adjacent normal tissue, or acquisition of a particularly favorable mutation in the cancer population, which allows proliferation causing late-term metastases^7^.

The unique eco-evolutionary dynamics of small populations have recently been recognized and investigated primarily through observations during Anthropocene extinctions^8–10^ and conservation of endangered species^11–14^. The involvement of population size in shaping fitness of individuals in the population was recognized already by Darwin^15^, and later described in detail by Allee^16,17^. However, the specific underlying mechanisims^18^ responsible for such “Allee” effects have only recently been clarified through field observations, experimental systems^19,20^, and mathematical formulation. Furthermore, the role of small population dynamics in cancer populations, visible for example in thresholds of inoculum size to initiate in vitro tumor cultures^21^ and in vivo experimental tumors, have been increasingly investigated^22–25^. We propose that understanding the unique vulnerabilities of small populations to extinction as well as the key mechanisms controlling them, can potentially improve adjuvant therapy outcomes.

## Cancer treatment as extinction

Cumulatively, >99% of all species that ever lived^10^, many larger and more heterogeneous and spatially dispersed than cancer populations, have gone extinct. In popular culture, extinction brings to mind the final downfall of all non-avian dinosaurs roughly 66 million years ago, commonly misperceived to have vanished “overnight” following the catastrophic impact of a meteor^26^. The similarity of eradication of cancer to an extinction event is well recognized^27,28^, and current cancer therapy with reliance on application of toxic agents at maximum dose intensity and density of administration, arguably mimics a catastrophic ecological perturbation like the one associated with the termination of the Cretaceous period (Text Box 1).

While mass extinctions, such as the Cretaceous–Paleogene extinction, were undoubtedly dramatic and powerful^29^ episodes, which in geological time scales seem as though they occurred in the blink of an eye, in biological time scales these were much lengthier dynamical processes that could have extended to several millions of years. Yet the tendency to characterize extinction as an instant ‘event’ rather than a dynamical eco-evolutionary process, still remains deeply rooted, not only in popular culture, but also in current cancer therapy practices. Here, we explore extinction as an eco-evolutionary process and focus on investigations of multi-cause, multi-step dynamics of Anthropocene extinctions (Text Box 2 and Text Box 3) as a potential model for adjuvant and neoadjuvant therapies in BC.

Box 1—Conventional cancer therapy: magic bullets and moving targets A central paradigm of 20th century medicine has been the notion of “magic bullets”, a term initially coined by the renowned medical scientist, Paul Ehrlich^79,80^. The concept of a “magic bullet” is based on the proposition that it should be possible to exclusively target and kill specific disease-causing agents (such as microbes or tumor cells) without harming the body itself. A notion that rapidly gained supported with, among others, the development of antibiotic “magic bullets” in the mid-20th century and, more recently, the selective control of HIV using a combination of antiviral drugs. Cancer therapy strategies often invoke an analogy to infectious disease in its ongoing endeavor to design antibiotic-like drugs that can specifically target each cancer type (“one drug, one disease”). Undoubtedly, this approach has been remarkably successful as evidenced by the steadily increasing number of agents available to treat localized and metastatic cancers.

*Ultimately however, most common metastatic cancers still remain fatal, and microscopic residual cancer populations typically survive multiple cycles of treatment even with highly cytotoxic agents. Why?*

One limit is host toxicity. Nearly all cancer treatment agents have potentially fatal toxicities that constrains administration to some “maximum tolerated dose” (MTD). Many investigators hypothesized current treatments could be converted to “magic bullets” if their doses could be increased. However, novel strategies, such as bone marrow transplants, that allow for significantly increased drug administration did not produce cures. Similarly, combination therapies often improve response compared to monotherapy, but resistance almost inevitably emerges. This touches on the second limit which is the *eco-evolutionary* *context* of cancer, which make it possible for cancer populations to evade even the finest “magic bullets” by becoming moving targets through a process of “evolutionary rescue”.

Indeed, the high diversity of individual cancer cells, even with in microscopic populations, allows adaptations, including rapid evolution of resistance also when multiple agents are applied simultaneously. In addition, a key difference between cancer cells and bacterial or viral infections, is that in contrast to the later, cancer cells have access to the vast storehouse of information of the human genome, which permits multiple mechanisms for resistance through epigenetic changes in individual cells. Furthermore, resistance strategies can also emerge from population-level dynamics produced by interactions between different cancer cells (*a.k.a.*, aggregation effects^43^), and/or interactions with host mesenchymal cells (*a.k.a.*, niche engineering).

Thus, a fundamental barrier to the “magic bullet” paradigm of modern medicine is *the eco-evolutionary context of cancer,* in which treatment and cancer cell proliferation take place on similar time scales, permitting consistent “evolutionary rescue”.

Box 2—Anthropogenic extinction of the passenger pigeon A classic example of an Anthropogenic extinction is the loss of the passenger pigeon, which only two centuries ago was possibly the most abundant bird on the planet. From a population estimated at ~4 billion individuals in the 19th century, the last know individual died in 1914, less than one century later. Initially, mass habitat destruction of hardwoods and mass hunting, were responsible for critically reducing population sizes. Although this “first strike” significantly reduced the passenger pigeon population, as well as progressively fragmenting it into even smaller weakly connected subpopulations, this in itself cannot be held responsible for the ultimate eradication of the species. Because the pigeon relied on large flocks to avoid predators, as a consequence of decreasing subpopulation sizes, these became increasingly more susceptible to predation. In addition, because the passenger pigeon also relied on social flocking for foraging and searching for community breeding sites, susceptibility increased even further. Inevitably, the combination of these additional factors led local passenger pigeon populations to even further decline, most probably also accompanied by additional fragmentation creating even smaller weakly connected subpopulations, and so the process continued in an accelerating rate. Hence, a snowball effect readily emerged as populations became smaller, and together with the increasing sensitivity to predators and loss of foraging and breeding sites, a self-reinforcing positive feedback cycle, also known as an “extinction vortex”, was created. As these destructive mechanisms became dynamically intertwined, it would have been practically impossible to determine which of the drivers was singlehandedly responsible for the final extinction.

Box 3—Intentional Anthropogenic extinction—the Galapagos goat Eradicating a cancer population can be viewed as an intentional Anthropogenic extinction process. A rare intentional extinction—termed Project Isabela—was carried in the Galápagos Islands in the late 20th Century. At that time, the feral goat population in the Isabela, Santiago, and Pinta islands rapidly increased about 250,000 individuals resulting in damage to the environment and threats to native species. This resulted in a world-wide consensus that the feral goat population must be eradicated to protect this World Heritage Site. Initial strategies employed sharpshooters armed with automatic weapons traveling in trucks and helicopters. This carnage eradicated ~90% of the goat population. However, “resistance” emerged as individuals and small groups of goats learned to escape into the forests upon hearing approaching helicopters. As this population increased, Project Isabela was required to adopt a new strategy. “Judas goats,” sterilized females coated with hormones and wearing a radio-tracking device, were released into regions known to contain resistant populations. They joined the small surviving groups allowing hunters to locate and kill them. The Galapagos goat was declared extinct in 2005.

The Galápagos goat extinction illustrates a relatively simple multi-step, multi-cause Anthropogenic extinction. The population’s initial decline was caused by a deterministic perturbation that predicably decreased the size, spatial distribution, and diversity of the goat population. This “first strike” produce a “race to extinction” but did not eradicate the population a resistant phenotype emerged producing “evolutionary rescue”. Note that, once resistance developed, continued application of the initial perturbation was futile. However, a new strategy took advantage of the extinction vulnerabilities of the surviving population. Thus, the release of a small number of Judas goats pushed the survivors into extinction. Note the Judas goat strategy, while ideal for the second-strike dynamics, would have been far too small to reduce the large initial large goat population just as the first strike strategy was ineffective once an adaptive strategy emerged in the surviving goat population.

### Lessons from Anthropocene extinctions

In the Anthropocene era, extinction rates are estimated as being 3–4 orders of magnitude higher than prior background extinctions due to a combination of human induced threats and stressors, including habitat degradation and destruction, overexploitation (e.g., fishing and hunting), chemical pollution, introduction of invasive species, spread of diseases, and climate change.

Although it is well recognized that each threat can trigger population decline and increase the risk of extinction on its own, the Anthropocene era has revealed that the characteristic co-occurrence of multiple stressors in this era, is in itself a major factor that is driving present day extreme extinction rates. Indeed, overlapping stressors frequently become intertwined in their disruptive effects, such that their combined effect may be greater than the sum of their parts (e.g., see Text Box 2). In such cases, this elicits self-reinforcing dynamics where extinction risk rapidly increases. In practice, attempts to reverse the decline of natural populations once they have entered a path to extinction frequently fail, particularly when management efforts focus only on single discrete stressors and not on their interactions.

Another common phenomenon is the recruitment of secondary indirect stressors into later stages of the extinction process; i.e., stressors that may be disconnected from the original drivers of decline, and/or ones that under normal conditions would not be labeled as indisputably harmful to the population. Yet, during a period of massive disturbance populations can becomes susceptible to new factors. For example, a population that is experiencing gradual habitat loss may become increasingly vulnerable^30–32^ to invasive species, predators, or disease. Hence, drivers of population decline, may facilitate the contribution of additional stressors, which synergistically lock endangered populations onto a downward trajectory of collapse via self-amplifying effects, leading populations to their extinction. The term ‘synergistic’ describes the simultaneous action of multiple distinct processes (extrinsic threats or intrinsic biological traits) that together have a greater total effect than the sum of each of their individual effects alone. Thus, in nature, *the final extinction of small populations is characteristically driven by synergistic effects of several perturbations, none of which, on their own, would have achieved this effect*.

### Extinction as an evolving process

Anthropogenic extinctions of large, heterogeneous and widely dispersed populations (e.g., the Carrier Pigeon—Text Box 2) characteristically display two distinct stages^10^. The process eventually leading to extinction typically begins with one or more major disturbances, such as severe habitat degradation and/or introduction of predators, which lead to a drastic reduction of population size, heterogeneity, and spatial continuity, that shifts the population into a new state of high susceptibility where its risk of extinction due to chance fluctuations becomes considerably higher. Spatially, the population may have been separated into smaller relatively isolated subpopulations of survivors^9^, each of which is significantly more susceptible to random catastrophic events than the total population would have been had it remained unfragmented In addition, population structure and composition (i.e., demographically, functionally and genetically) is likely to be highly disrupted as well. As populations continue to decline, their susceptibility increases, and factors that previously played no role in driving this gradual collapse, may become stressors themselves due to the changing circumstances of the population.

Although such first strikes can have devastating effects on the population (Text Box 3), these extrinsic triggers of decline alone are usually not sufficient to fully terminate a population^33^. Rather, the first strike reduces the species to small and highly fragmented groups of survivors that are highly vulnerable to extinction caused by second strikes. Key lessons learned from Anthropogenic extinctions are: 1. Second-strike perturbations that produce extinction are nearly always different from those that caused the initial population decline (i.e., in the context of cancer treatment, they are resistant to the first strike agents). 2. Effective second-strike agent would often have very little effect on the population in its initial state when it was large^10^. 3. Second-strike perturbations tend to make populations ever smaller, reinforcing their own effect and those of other perturbations. This can readily lead to an extinction vortex with complex synergistic dynamics in which the relative contribution of individual stressors are difficult to disentangle from each other.

In prior studies, we have proposed these “First strike-second strike” dynamics may provide novel insights into treating widely metastatic cancers. Here, we focus on the dynamics of second strikes against small populations to provide novel insights into adjuvant and neoadjuvant BC treatment. This final slide to extinction facilitated by second strikes typically involves complex, synergistic eco-evolutionary forces characterized as an “extinction vortex”^34^.

### Components of the extinction vortex

#### Stochastic effects

All natural populations, including cancers, are subject to some level of stochasticity (unpredictable individual or environmental variability)^35,36^. Consequently, there is always some risk the population will randomly fall to zero due to chance. Spontaneous regressions of cancer, though rare, are indeed observed^37^. Importantly, this risk increases as the population declines so that even stable small populations^38^ can become extinct due to chance fluctuations alone.

The probabilistic nature of birth and death (*a.k.a.*, demographic stochasticity), can lead to deviations in the number of cells that die or proliferate in a unit of time, which deviates from the expected deterministic population averages due to chance alone. The source of individual variation can be paralleled to flipping a coin for each individual in the population. When populations are large individual variation has little impact on the population size and population growth remains close to its expected deterministic rate (e.g., with many coin flips, the number of heads and the number of tails will be close to equal). However, as populations become smaller, random variation in the fates of individuals become more influential, introducing temporal fluctuations in population growth (e.g., with only a few coin flips, the skew between heads and tails can be substantial and is increasingly shaped by chance the fewer the coin flips). Hence, effects of demographic stochasticity scale inversely with population size. The smaller the population, the larger the random fluctuations in population size and the higher the risk of stochastic extinction.

In addition, environmental conditions can randomly fluctuate over time (*a.k.a.*, environmental stochasticity). In this case, the expected proliferation and mortality rates of the entire population vary with time (as if on some days the coin is weighted to give heads 80% of the time, and on other days 20% of the time). Such temporal environmental variability affects the entire population and, as a result, does not scale with size to the same extent as demographic stochasticity^39^. In the context of growing cancer, environmental stochasticity can result from fluctuations in blood flow that delivers substrate and growth factors as well as systemic treatment agents^40^. Intratumoral environmental stochasticity both influences and is influenced by the immune response, which can add cytokines to the interstitial space as well as predator-like T cells that can directly attack and kill cancer cells. For natural populations, environmental stochasticity is mitigated by having numerous subpopulations each of which experiences environmental ups and downs out of sync. A single, small subpopulation bears the full brunt of environmental fluctuations and may be driven to extinction by environmental stochasticity alone.

Although demographic and environmental stochasticity act in different density-dependent ways^35,36^, their negative effects increase as the size of populations and the number of subpopulations decline, respectively. Thus, the probability of population persistence diminishes at an increasing rate as populations become smaller.

#### Allee effects

Because individuals can produce far more offspring than can possibly survive, there is an ecological struggle for existence built into Darwin’s theory of natural selection. Hence, at some point as the population nears its “carrying capacity”, the proliferation rate of individuals must decline as the population size increases because of growing intraspecific competition for space and resources. However, Charles Darwin also observed that, for many species when population sizes are still small and very far from their upper limiting carrying capacity, the threat of predation and competition seemed to also decline as the population became larger. In these cases, proliferation rates increased with population size. Along similar lines, in the 1930’s the ecologist Warder Clyde Allee found that survival and fecundity of individuals frequently declined in smaller populations, significantly increasing the risk of their extinction^17,41,42^. A related dynamic, termed “aggregation effect”^43,44^, finds isolated individuals are often more adversely affected by a perturbation than when they are in a group (e.g., a herd). Indeed, for many species favorable interactions among individuals, such as group defense, co-feeding, ecological engineering, and group foraging, have been found to increase with population size when populations are relatively small^20,23,45^.

Although vulnerability to stochastic effects is a universal property of small populations including cancer, the role of Allee effects is typically species-specific. Cancer populations typically arise from a single cell or small number of cells; and, as in nature, individual cancer cells within a population remain the evolutionary “unit of selection”. However, population-level interactions among these cells (e.g., angiogenesis^40^, matrix remodeling^46^, environmental acidification^47^, metabolic “cooperation”^48–50^), resulting in Allee effects have been well documented^22,23,25,49,51^. Although localization of mates is a significant factor in sexually reproducing small populations, asexual reproduction in small populations makes it harder to purge the accumulation of deleterious genetic changes (“Genetic Allee effects”)^52^.

These Allee and aggregation effects alter the ability of small and disturbed populations to adapt to the host immune response and to additional perturbations in the form of adjuvant cancer treatments.

In addition, as populations become smaller and more disturbed, their chances of fadeout through stochastic fluctuations increases, and the fluctuations themselves are amplified. Hence, a synergy emerges between stochastic effects and Allee effects that places the population on a transient towards extinction, i.e., the “extinction vortex”.

## Adjuvant/neoadjuvant therapy as an Anthropogenic extinction

**Fig. 1:**
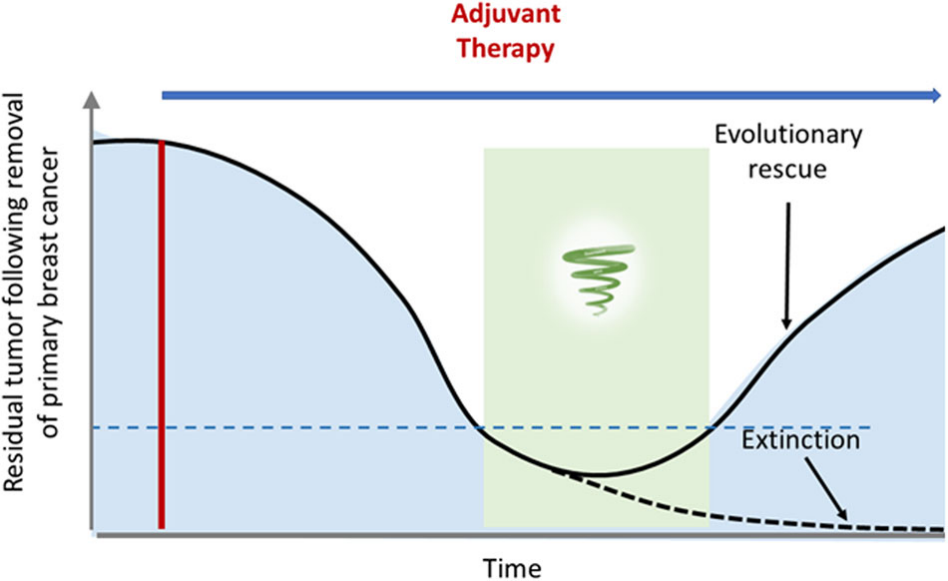
Evolutionary dynamics of adjuvant therapy. Initial treatment causes a decline in the global tumor population. This sets off a “race to extinction”. If the initial treatment imparts sufficient evolutionary force, the population will be forced into an extinction vortex and become extinct with no additional perturbations. However, if resistance develops and manages to successfully produce a growing cohort before fading out, a surviving population could permit “evolutionary rescue” such that the tumor recovers and proliferates, eventually forming a clinical metastasis. The period of time in which the tumor is in the extinction vortex represents an opportunity to add new treatments for accelerating the extinction process and for reducing the risk of evolutionary rescue.

The course of adjuvant or neoadjuvant therapy for the treatment of clinically undetectable metastatic disease is shown in Fig. 1. The initial treatment radically reduces the size of the tumor, leaving one or more small undetectable sub-populations of cells that are highly vulnerable to extinction. Additional treatments increase the chances of pushing these clusters into an extinction vortex, where the remaining populations is losing cells, heterogeneity, individual fitness and spatial connectivity faster than it can regenerate these. A combination of treatments may accelerate this process, to ensure that the population remains locked in the extinction vortex, and continues to decline until extinction. This represents successful adjuvant treatment. At this point, the only way for the population to escape its fate is through “evolutionary rescue”, where a subpopulation has, or can develop, resistance prior to extinction, and can significantly express it under these circumstances, to allow a surviving cohort to breakout of the transient of deterioration it is locked into, eventually leading to progression with the formation of metastases. This can be described as a “race to extinction” because while the “risk” of extinction is rapidly increasing, the prospect of rescue through resistance still exists. However, within the extinction vortex, this prospect rapidly decreases due to genetic drift [Ref: BOOK: “The logic of chance: the nature and origin of biological evolution” by Eugene V. Koonin, editor., FT Press, 2011] and decreasing capacity to generate a cohort of sufficient size.

Adjuvant therapy has been extensively studied in women with primary BC and both extinction and evolutionary rescue are observed. A large analysis^53^ following widespread adoption of adjuvant therapy, found, for women under age 50, that adjuvant chemotherapy delayed recurrence (41.1% recurrence at 15 years vs 53.5% for control) and reduced mortality (32.4% at 15 years vs 42.4% for control). For women between ages 50 and 69, the benefits were somewhat lower for both time to recurrence (53.4% vs 57.6% control) and mortality (47.4% vs 50.4% control).

More recent clinical investigations have largely focused on optimizing treatment for three clinical subgroups: ER^+^HER2^−^, HER2^+^, and triple-negative BCs. The cumulative experience in a cohort of 100,000 women^54^ clearly indicates that adjuvant chemotherapy can cause extinction in 20–30% of BC subtypes but the majority of microscopic BC metastases undergo evolutionary rescue and progress to clinical disease.

An obvious strategy to improve outcomes is increasing the evolutionary force of the initial adjuvant therapy can increase the probability of extinction. For example, the combination of Trastuzumab and Emtansine through an antibody-drug combination increased the percentage of patients who were disease-free at 3 years to 88% compared with 77% for Trastuzumab alone^55^. However, that combination of drugs in an adjuvant or neoadjuvant therapy provides no increase in the probability of extinction and can increase toxicity^56^.

Figure 1 demonstrates the potential value of intervening at the evolutionary inflection point following initial administration of adjuvant therapy. Here additional treatments applied to exploit the dynamics of the extinction vortex can potentially increase the probability of extinction. Here, there are 3 key lessons:

### Time is of the essence

When not subjected to any external perturbations, small cancer population will both expand and, by occupying new niches, genetically diversify^57^. During this time, the probability that any given perturbation will cause extinction significantly declines. Thus, long delays in the application of adjuvant therapy or between treatments during therapy increase the probability that resistant populations will emerge prior to or during the treatment.

Historical data originating from large trial using anthracycline-based perioperative chemotherapy suggest no clear differences in outcomes between patients receiving neo or adjuvant therapy for BC^58^. The clinical experience varies with tumor type and treatment protocol, multiple studies have found that more prolonged time between surgery and initiation of adjuvant therapy is significantly associated with decreased overall survival^59^ in ovarian and BC^60,61^ and has been demonstrated in multiple pre-clinical studies^62,63^. In general, these dynamics suggest the probability of eradicating small metastatic cancer populations decreases as population size increases so that treatment should begin as soon as possible following diagnosis.

### Small population extinctions usually require multiple and sequential perturbations

Although single agents can drive small cancer populations to extinction, clinical experience demonstrates that multiple agents generally improve outcomes. The addition of other drugs is thus beneficial. This is the case for aggressive BCs as dual HER2 blockade and simultaneous combination of chemotherapy agents with distinct mechanisms of action lead to broader cell destruction than each individual agent alone^64^. Nevertheless, clinical data demonstrate that rational sequencing of agents is also a key component of development of treatment regimens. To exemplify, the sequential treatment with taxanes after treatment with anthracycline-based regimens improves outcomes in patients with early BC are now standard for patients with more-aggressive BCs^65^. But what is the evolutionarily optimal strategy for combining more than one drug?

The synergistic, self-reinforcing dynamics of small populations in the extinction vortex are dependent both on the type of perturbations and their temporal association. For example, in cancer treatment, two drugs given simultaneously often result in greater tumor cell death than when each drug is administered individually. Indeed, response rate to neoadjuvant treatment with taxane is higher when combined with carboplatin^66^. However, administering both drugs when the cancer population is large and heterogeneous also maximizes the probability that a subpopulation resistant to both drugs will be present. This may be one of the reasons the latter two agents have not improved survival in patients with early BC. Assuming most cancer populations have weak Allee effects, the population will begin to rebound (though often sluggishly) once only resistant individuals persist. Increasing therapeutic pressure by adding more cycles is unlikely to result in improvement—if the initial combination of agents fails to produce extinction then continued administration of the same treatment to the resistant survivors is futile. Observations from the extinction emphasize the importance of sequential perturbations in small populations because the reduction in population size from an initial stressor increases its vulnerability to subsequent insults.

Most adjuvant/neoadjuvant therapies in BC use more than one agent, usually administered concurrently. However, Mavroudis et al.^67^ compared epirubicin and docetaxel given sequentially (four cycles of epirubicin followed by four cycles of docetaxel) or simultaneously for six cycles and found a small but non-significant increase in disease-free survival in the sequential group (92.6% vs 88.2%). A meta-analysis of Phase III randomized trials by Shao et al.^68^ concluded that, for combined anthracyclines and taxanes, sequential adjuvant chemotherapy for BC provides a significant benefit in both disease-free survival and overall survival over concurrent regimens. Indeed, simultaneous administration of taxanes and anthracyclines has been virtually abandoned in clinical practice owing to increased toxicity and no clear improvement in clinical outcomes.

In HER2^+^ BC, trastuzumab (Herceptin) increase the overall cell death rate by directly targeting HER2. However, it also elicits an immune response and the subsequent intratumoral lymphocytic infiltration predicts treatment efficacy^69–71^. Interestingly, as the targeted response will occur immediately, but the immune response requires some time to develop, trastuzumab therapy effectively applies a sequence of perturbations to the cancer, which may account for its superiority to other tyrosine kinase inhibitors. The potential role of sequencing treatment is supported by observations that neratinib, an irreversible pan-HER kinase inhibitor, when administered following trastuzumab and chemotherapy, significantly reduced relapse in patients with high-risk early BCs^72^.

### Most extinctions involve ecological disruption

Nearly all adjuvant therapy protocols focus on demographic perturbations. That is, they directly attack individual cancer cells to alter the proliferation and death rates of the population. This is a reasonable strategy since it results in population decline that, if maintained over time, will inevitably produce extinction. However, this strategy can fail in a heterogeneous population in which drug sensitivity is variable so that an initial decline may select for resistant phenotypes, which could survive and repopulate the tumor. As noted above, ecological disruptions apply an entirely different form of “selection” pressure to the entire population which, among others, also increase the likeliness these phenotypes fade-out before successfully establishing a growing cohort. In the context of small cancer populations, this strategy might include antiangiogenic agents or drugs that selectively target microenvironment conditions such as hypoxia activated prodrugs. The tumor ecosystem contains not only myriad cells with distinct evolutionary capabilities but also a multitude of immune cells that have the potential to eliminate small cancer populations. From the clinical standpoint, manipulation of tumor microenvironment has shown promising results for the treatment of triple-negative BCs. In the I-SPY 2 and the Keynote 522 trials, stimulation of the tumor lymphocytes by pembrolizumab significantly increased pathological responses when combined with paclitaxel and carboplatin after treatment with doxorubicin and cyclophosphamide^73,74^. Interestingly, activation of co-existing immune cells by pembrolizumab has been associated with numerically longer recurrence-free survival in preliminary analysis, suggesting that long-lasting changes in the tumor ecosystem lead to eradication of small or subclinical small cell population.

As interactions between the immune systems and cancer cells begin to unveil, new data also emerge suggesting that alternative ways of sequencing and administration of treatments could lead to regression of populations. Interestingly, pre-clinical data supports that modulation of tumor microenvironment towards cytotoxic states renders chemotherapy more effective against cancer cells^75^. Nonetheless, administration of metronomic doses of chemotherapy has also shown synergistic effect with developing immune treatments^75^. Collectively, these data suggest that interplay between ecological pressures are more complex than the conventionally accepted paradigm of achievement of MTD regimen. We bring attention to the caveats in the realm of developmental therapeutics for BC. The current paradigm may erroneously assume that a catastrophic event to a small population necessarily represents a devasting event to an entire ecosystem (i.e., increased patient toxicity). This is not necessarily the case. To illustrate HER2-primed dendritic cell vaccines have shown to lead to tumor regression has with low absolute risk of high-grade adverse events^76^. Furthermore, the development of treatments under the premise of improved outcomes at MTD regimens assumes uniform exposure of cancer cells with increased dosages. A cancer agent may not eradicate a small population of cancer cells as a function of poor drug delivery, which is inherent to agent and or the tumor type^64^. Alternative treatments may lead to improved access to small populations facilitating broader changes in the ecosystem.

## Conclusion

The novel eco-evolutionary dynamics of small populations suggest new strategies for adjuvant therapy that are predicted to improve outcomes. These include: 1. Initiation of treatment as soon as possible following diagnosis. 2. Treatment should cycle different agents or combinations of agents ideally with different mechanisms of action and resistance. Each cycle should be relatively short (e.g., every 2 weeks) and delay between cycles should be minimized as dose density has been associated with improved outcomes^77^. 3. Ecological agents such as passive and active immune strategies should continue to be considered in future clinical trials. Although antiangiogenic agents have had limited effect in prior BC clinical trials, perturbations in blood flow might be effective when applied in an evolutionary sequence with other agents.

Although this is clearly a departure from standard oncologic practice in adults, we note these dynamics are likely observed in the empirically derived, multi-step curative treatment in pediatric acute lymphocytic leukemia.

Finally, treatments applied during the extinction vortex do not necessarily have to include only those that have demonstrated benefit in large of the same cancer. That is, even agents to which many of the cells are resistant, may have a significant negative effect on a segment of an already small population simply because they must expend additional resources to deploy the molecular machinery of resistance. Once a small cancer population is in the extinction vortex, *any* drug that applies stress to the remaining cells is highly likely to be of value.

Adjuvant treatment of residual BC after intense neoadjuvant therapies for both TN and HER2^+^ BCs epitomizes this new paradigm. Adjuvant treatment with capecitabine and T-DM1 significantly reduces the probability of TNBC and HER2^+^ BCs, recurrence, respectively^55,78^. These results further corroborate the notion that both alternative and sequential insults to small BC populations leads to clinically relevant improved outcomes.

Thus, although the search for “magic bullets” should continue, we propose that currently available drugs can be strategically combined that significantly increase the chance of complete cure. Even when none of the drugs is individually curative and all of them given simultaneously is also not curative, an ecoevolutionary-informed sequence based on the dynamics of the extinction vortex can lead to successful eradication of the cancer population.

## Data Availability

No new or unpublished data were presented in this manuscript
